# Retracted articles in rehabilitation: just the tip of the iceberg? A bibliometric analysis

**DOI:** 10.1186/s40945-020-00092-w

**Published:** 2020-11-30

**Authors:** Marco Bordino, Elisa Ravizzotti, Stefano Vercelli

**Affiliations:** 1 Private Practice, Vercelli, Italy; 2 Department of Neurorehabilitation Sciences, Istituto Auxologico Italiano, IRCCS, Milan, Italy; 3 Physical and Rehabilitation Medicine Unit, Istituti Clinici Scientifici Maugeri, Institute of Veruno, IRCCS, Gattico-Veruno (NO), Italy

**Keywords:** Retracted publication, Rehabilitation, Physiotherapy, Research misconduct

## Abstract

**Background and aim:**

The volume of withdrawn publications in scholarly disciplines has grown steadily, but there is little awareness about this issue in rehabilitation. The aim of this study was to analyze the extent of retracted articles pertaining to rehabilitation.

**Methods:**

Retracted articles were searched in 4 different bibliographic databases from their inception to April 2020: PubMed, Web of Science, WikiLetters and Retraction Watch. Three independent reviewers assessed the relevance of the retrieved articles to the rehabilitation area.

**Results:**

Of 280 rehabilitation-related publications retracted between 1984 and 2020, 83 (29.6%) were published in 55 full open access journals and 197 (70.4%) were published in 147 traditional, non-open access or hybrid journals. In the last 10 years (2009–2018) there was a significant steady increase in both the total number of retractions (*p* < 0.005; *r* = 0.856; *R*^2^ = 0.733) and retraction rate per year (*p* < 0.05; *r* = 0.751; *R*^2^ = 0.564). However, the number of retractions represents a very small percentage (~ 0.1%) of the overall volume of publications in rehabilitation.

**Conclusions:**

Our data indicate that the number of retracted articles in rehabilitation is increasing, although the phenomenon is still limited. However, the true prevalence of misconduct may go unnoticed due to the large number of low-quality journals not indexed in the searched databases. Physiotherapists should be aware of the danger of misleading information originating from withdrawn publications.

**Supplementary Information:**

The online version contains supplementary material available at 10.1186/s40945-020-00092-w.

## Background

In recent years, there has been an increased focus on the retraction of publications in several scientific fields. Retraction is defined as ‘a mechanism for correcting the literature and alerting readers to articles that contain such seriously flawed or erroneous content or data that their findings and conclusions cannot be relied upon. Unreliable content or data may result from honest error, naïve mistakes, or research misconduct’ [1]. Retractions are an important mechanism to preserve the integrity of the literature [2–4], but they also represent a failure of the peer-review and publication processes. The retraction of an article is often related to scientific fraud and misconduct, which damage the whole scientific community as well as the institution and department from which the withdrawn manuscript originated [5–7]. However, retracted articles are potentially damaging even in cases where there is no ill intent and the retractions are due to errors rather than to misconduct.

When a scientific article is withdrawn after publication, the journal issues a retraction notice. The term “retraction” refers to the publication subsequently withdrawn, while “retraction notice” indicates the publication of a notice of withdrawal associated with a previously published article [8]. The latter often also indicates the causes that led to the retraction and the authorities responsible for the retraction initiative (authors, editor, and publisher).

Until the 1990s, the implications of the withdrawal of scientific publications aroused little focus, as evidenced by the lack of literature on the subject and guidelines available. In 1998, Budd et al. [9] conducted a retrospective analysis of all withdrawal notices in the scientific literature, identifying 235 manuscripts from 1966 to 1997. The authors were the first to emphasize the frequency of published retraction notes, identify their causes, and report concern that withdrawn articles could continue to be cited despite being retracted [9, 10]. The awareness of this issue among journal editors increased particularly after 2009, when the Committee on Publication Ethics (COPE) published the first set of guidelines to editors on the issuing of retractions [11]. The phenomenon has since grown considerably: before 2003 there were less than 50 withdrawals per year [12]; in 2009, the number was five times higher; and in 2013, it was eight times higher [13]. In December 2019, the COPE retraction guidelines have been updated [1]. However, there are different views on the matter. Some authors see the continuous annual increase in retractions as an urgent problem, despite the fact that articles retracted still represent only a small percentage of the scientific literature [3, 14]. Others argue that the frequency of retractions has grown more than the increase in the number of publications [15–17], but that it is uncertain whether this increase is due to an increased rate of submissions warranting retraction or to a higher awareness by publishers and reviewers of the issue. Though the percentage of articles retracted in the medical/health sector is higher than in other disciplines [18], it has been hypothesized that only a fraction of cases of misconduct are identified and publicly disclosed [19, 20].

Articles may be withdrawn for a variety of reasons, and their increase may not actually reflect a scientific integrity crisis. Anyhow, this phenomenon is particularly important in the biomedical field where flawed publications may serve to mislead other scientists, clinicians and patients. In most cases, retracted articles continue to be cited as being valid and negatively affect the development of the scientific knowledge [3, 10, 14–17, 19–32]. The concern is particularly serious when such publications may influence patient choices with regard to treatment options [33, 34].

Previous studies on retracted articles have focused on the biomedical literature as a whole or on specific medical fields, but no study has examined in-depth, to our knowledge, the extent of the problem in the area of rehabilitation and physiotherapy. Therefore, the aim of this study was to estimate the bibliometric volume of retracted articles pertaining to rehabilitation. The results will be useful to characterize the landscape of article retractions, in order to sensitize professionals to a more responsible scientific production and to the need to stay updated on the issue of new retractions for a critical and more-aware use of the literature.

## Methods

A bibliometrics analysis of retracted publications in the rehabilitation area from four databases was performed. The Preferred Reporting Items for Systematic Reviews and Meta-Analyses (PRISMA) Statement was used in the preparation of this manuscript [35].

### Search methods

Two authors independently searched the four databases (PubMed, Web of Science, WikiLetters, and Retraction Watch) from their inception until the last date of the search on 21st April 2020. Specific terms and strategies were generated for each database in order to identify retracted scholarly articles. First, two scientific biomedical databases were used. In PubMed, publications were identified by searching for the free-text terms retraction note OR retraction notice, with no filter/limitation imposed on publication date or topic of the articles. Web of Science (WoS) was searched with the title “retraction of” and refined with the filters: category “rehabilitation” and type of document “retraction”. Data were also extracted from two retractions-specific databases. WikiLetters in its WL-RETRACTED branch was searched by inserting 75 different search terms relating to rehabilitation. Retraction Watch was searched setting the following filters: Subject “(HSC) Medicine - Rehabilitation” and Nature of Notice “Retraction”. The search employed in each database is shown in the Additional file 1. Duplicates were then removed. PubMed publications were cross-referenced to the ones found in the other databases. All the relevant articles not present in PubMed were then added to our database.

### Eligibility criteria

Titles and abstracts were first screened to identify publications that met the following criteria: (1) related to physiotherapy and/or rehabilitation medicine, (2) published in English, and (3) with a retraction notice or other editorial notification vehicle explicitly issued and available. The articles were also perused in a wide-knit preliminary fashion, in order not to lose potentially relevant records. Full text publications were then obtained and carefully re-assessed for eligibility by three independent reviewers to establish their relevance to the rehabilitation area. Only results that obtained approval from at least 2 out of 3 reviewers were included in the study. The judgment of relevance to rehabilitation and physiotherapy was independent of the type of journal, i.e. articles belonging to other scientific sectors could be included if deemed of interest for clinical practice in rehabilitation.

Retractions referred to letters, comments, abstracts, conference proceedings/papers, posters, and erratum, were excluded. We also excluded “expression of concern” notices. These latter are usually issued by editors when they believe that there is sufficient evidence to question published data, but not to retract an article.

### Data extraction

Retracted articles were imported into a spreadsheet and electronically linked to their retraction notice. The year of retraction of each article was identified on the retraction notice. The following data were extracted from eligible full publications: title, area (musculoskeletal, neurology, cardiopulmonary, gerontology, continence and women’s health, orthopedics, pediatrics, ergonomics, occupational health, oncology, sports/physical activity, others) and topic (rehabilitation, outcome measures, surgery, drugs, biomechanics, epidemiology, diagnostic, modalities, physical activity, complementary/alternative medicine, others) of the article as classified in the PEDro database [36], type of study (systematic review, scoping review, meta-analysis, randomized-controlled trials, methodological, observational, letter/note, protocols, others), year of retraction, name and type of the journal, and the source database. The official journal’s websites were searched in their ‘Submission Guidelines’ and ‘Information for Authors’ sections to identify whether the journal was a pure Open Access (OA) or a Non-Open Access (N-OA). Although most traditional journals are hybrid, i.e. they offer the double option of OA or subscription, we classified them for the purpose of this study as N-OA.

### Data analysis

For all analysis, SPSS 20.0 statistical software was used. We first performed a descriptive bibliometrics analysis to estimate the annual volume of retracted articles, expressing data as mean ± standard deviation (SD), median and interquartile range (IQR), numbers, and percentages. We then performed a linear regression analysis to evaluate the correlation between the number of retractions and publication year. R-squared (R^2^) was used to indicate the percentage of the variance in the number of retracted articles that could be explained by publication year. The statistical significance of the regression model was set at *p* < 0.05. The model was then compared to the entire volume of publications in the rehabilitation area, which was estimated through the PubMed search with the following term: “Rehabilitation”[Mesh]. The retraction rate per 10,000 publications/year was also calculated, and Pearson’s correlation coefficient test (r) was used to evaluate the proportion of retracted papers over the years in the last decade.

## Results

**Fig. 1:**
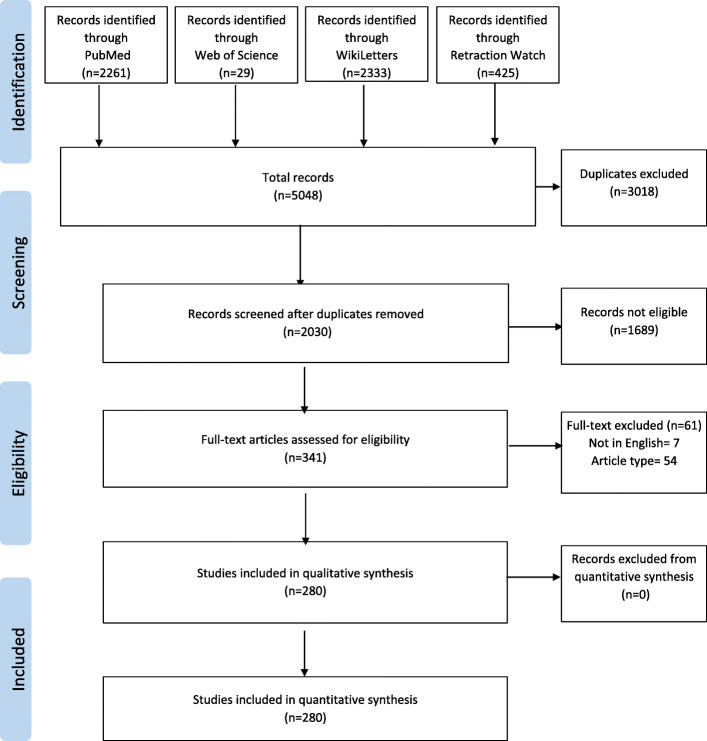
PRISMA flow diagram outlining the study selection process

Our search yielded 5048 results between 1984 and 2020. After removal of duplicates and articles not related to the topic (as deemed by three independent reviewers), 341 retracted articles were reviewed on a full text basis. There was a high agreement between the researchers: 266 (78%) records obtained three positive assessments for inclusion. Of the selected publications, 280 met our inclusion criteria (Fig. 1). Most of the retractions were identified in PubMed (*N* = 250; 89%), the remaining were available only in WikiLetters (*N* = 17; 6%), WoS (*N* = 8; 3%), or Retraction Watch (*N* = 5; 2%).

**Fig. 2:**
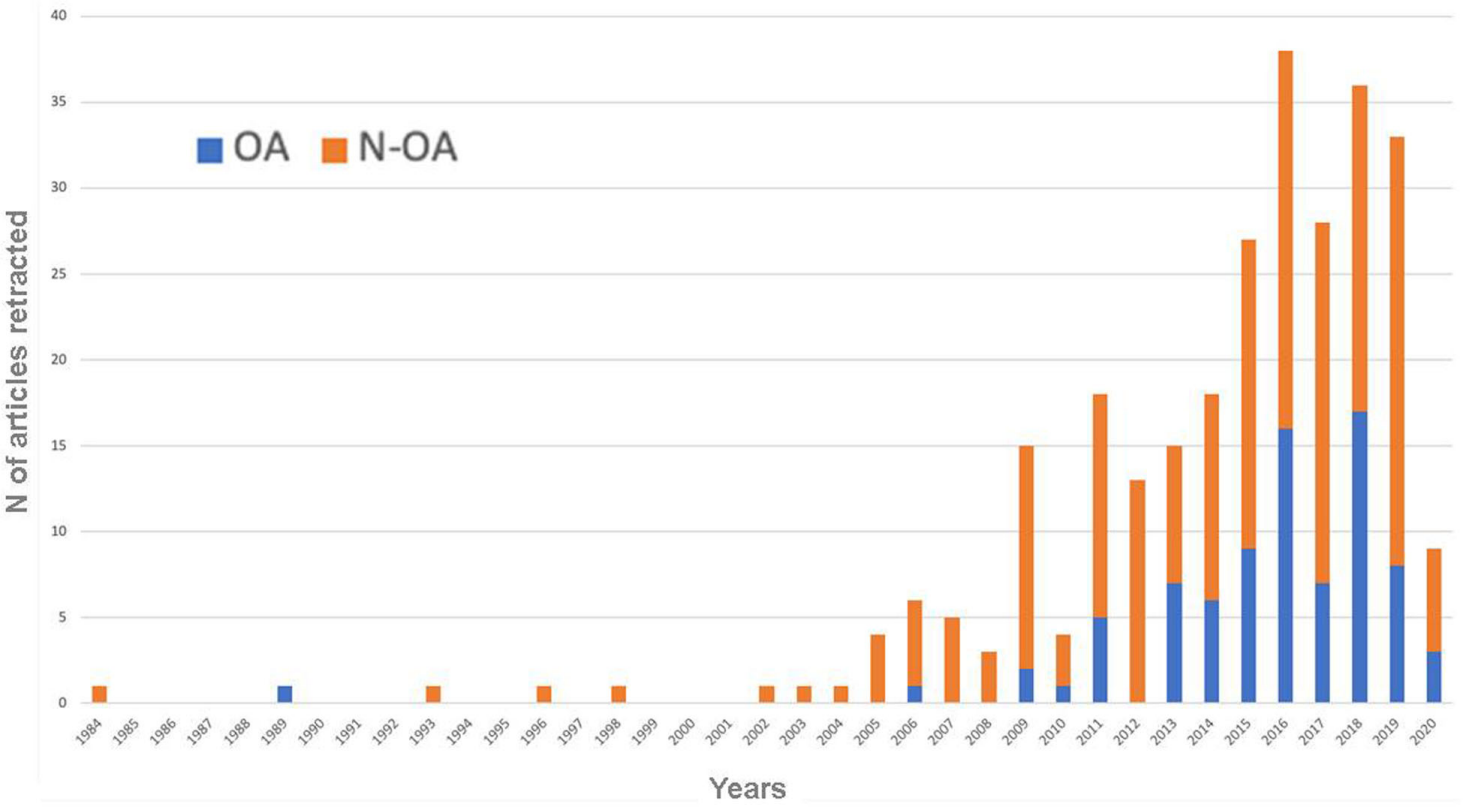
Distribution of retracted articles across the period 1984–2020. Legend: OA, open access journals; N-OA, non full open access journals

It was possible to determine the year of retraction for each of the included articles. The first retraction notice in the rehabilitation area was issued in 1984. Whereupon, there was modest or no growth up until 2009, when a marked increase occurred and this trend continued until a peak in 2016 with 38 articles retracted (Fig. 2).

Given that the length of median article lifetime between publication and withdrawal is about 15 months [37], we focused on the period 1984–2018 to assess the trend in number of withdrawals, excluding years 2020 (*N* = 9) and 2019 (*N* = 33). Most publications have been withdrawn in the last 10-year period, with a further net increase observed in the last 5 years (Table 1).

**Table 1:** Distributions of retracted articles across different time-periods. Results from 2020 and 2019 were excluded

	All (1984–2018)	Last 10 years (2009–2018)	Last 5 years (2014–2018)
**Total counts (%)**	238 (100%)	212 (89%)	180 (76%)
**Mean (SD)**	6.8 (10.9)	21.2 (10.8)	29.4 (8.0)
**Median (IQR)**	1 (0–13)	18 (14.5–30)	30.5 (22.5–37)

The regression analysis regarding the interval period 2009–2018 showed a strong correlation (*r* = 0.856) between the year of publication and the number of retractions (*p* < 0.005). The proportion of total variation (R^2^) explained by the independent variable was 0.733.

**Fig. 3:**
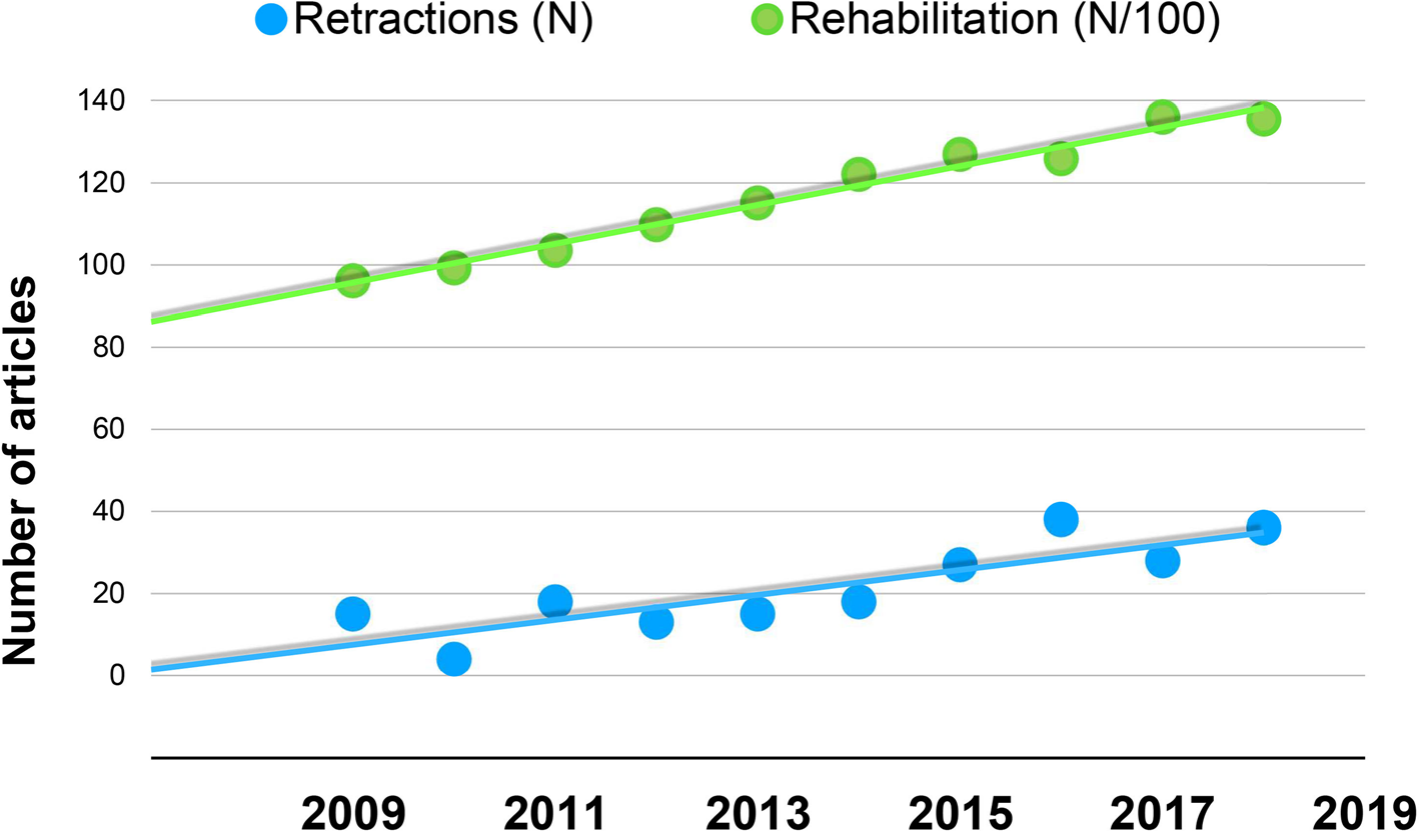
Trends in publication and retractions in the rehabilitation area in the period 2009–2018. Green dots represent all articles published in PubMed (N/100), and blue dots represent retracted articles included in this study. Linear trend lines are also shown

This strong linear trend was very similar to the growth of entire publications in the rehabilitation area (*p* < 0.005; *r* = 0.990; *R*^2^ = 0.980) in the same period (Fig. 3).

**Fig. 4:**
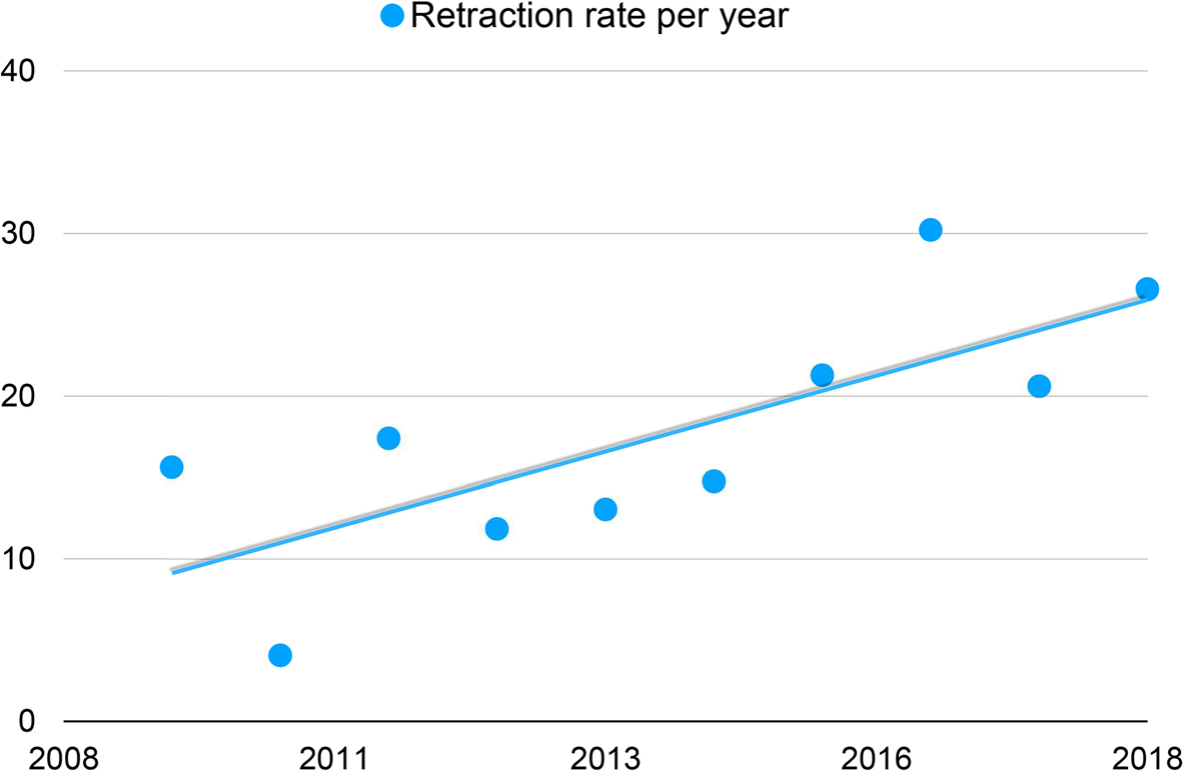
Retraction rate per 10,000 publications in the period 2009–2018

In 2009, a total of 9609 articles were published in the rehabilitation area according to PubMed, yielding a retraction rate of 15.6 per 10,000 publications (15 out of 9609). This rate increased to 26.6 per 10,000 publications (36 out of 13,555) in 2018, with a mean of 17.5 (SD 7.5) for the entire period. There was a significant correlation between retraction rate and the decade 2009–2018 (*p* < 0.05; *r* = 0.751; *R*^2^ = 0.564) (Fig. 4).

The area, topic and type of the 280 studies retracted are resumed in Table 2.

**Table 2:** Distributions of area, topic, and type of study of retracted articles. ^a^A retracted article could be categorised in more than one area

Area^a^	N	Topic	N (%)	Type of study	N (%)
Musculoskeletal	143	Rehabilitation	55 (20%)	Observational	91 (33%)
Neurology	69	Drugs	47 (17%)	RCTs	65 (23%)
Sports/Physical activity	52	Surgery	36 (13%)	Scoping review	27 (10%)
Orthopaedics	38	Physical Activity	36 (13%)	Systematic review	18 (6%)
Gerontology	32	Diagnostic	18 (6%)	Meta-analysis	15 (5%)
Cardiopulmunary	9	Epidemiology	17 (6%)	Methodological	14 (5%)
Pediatrics	8	Complementary/alternative medicine	12 (4%)	Letter/note	1 (0.5%)
Continence and Women’s health	3	Outcome measure	11 (4%)	Protocols	1 (0.5%)
Oncology	3	Biomechanics	7 (3%)	Others	48 (17%)
Ergonomics	2	Modalities	4 (1%)		
Occupational health	2	Others	37 (13%)		
Others	94				
**Total**	**455**	**Total**	**280 (100%)**	**Total**	**280 (100%)**

The 280 retracted articles appeared in 202 different scientific journals over the entire period (see Additional file 2). The vast majority of journals issued only one retraction notice (*N* = 154, 76%), while a smaller proportion issued two (*N* = 31, 15%). There were 17 journals (8%) from which three or more articles were withdrawn. Of these, 8 journals had three retracted articles, 5 journals had four retractions, and 4 had as many as five retractions.

Overall, there were 55 (27%) OA and 147 (73%) N-OA journals listed. Among the N-OA journals, 111 (83%) were classified as hybrid and 22 (17%) as traditional journals. Of the 280 retracted articles, 83 (29.6%) had been published in OA journals, 167 (59.7%) in hybrid journals, and 30 (10.7%) in traditional N-OA journals (Fig. 2). The data focusing on the decade 2009–2018 (*N* = 212) are close to those for the overall study period: 70 retractions (33%) from OA and 142 (67%) from N-OA journals.

## Discussion

The aim of this study was to estimate the volume of retracted articles pertaining to rehabilitation and physiotherapy. To do this, we performed a thorough analysis of four databases, without limiting the results to specific journal indexes. The decision on which publications were relevant to rehabilitation was made by consensus. Consequently, some articles belonging to other scientific fields such as orthopedics, neurology or cardiology but considered relevant for clinical practice in rehabilitation were included.

The number of retracted articles in the rehabilitation area has shown a progressive annual increase, in line with other medical fields [38–43]. In particular, for the period 2009–2018 there was a strong linear trend of increase (*R*^2^ = 0.733). Several factors may have contributed to this growth. First, the greater awareness and attention of publishers, who are increasingly checking “further back” in their published archives to retract incorrect or fraudulent publications. The issue of scientific misconduct has become a priority for many international journals and editorial boards especially after the publication in 2009 of the COPE guidelines on how to handle retractions [12]. In 1999, only 38% of biomedical journals published or reported that they had a retraction policy [44]; in 2012, this percentage had risen to 65% [45]. Obtaining clear evidence of poor data integrity is often difficult to do [46–48], so it is not possible to retract the article immediately. However, by publishing an “expression of concern” notice they can quickly warn the research community about serious doubts regarding the published data, particularly in the early stages of the investigation, or about cases with ambiguous results. This notice is then reaffirmed or removed, as appropriate. Second, the tools used to detect cases of misconduct have improved: today many journals rely on plagiarism detection software or the Déjà vu database [49], as well as performing cross-checks on the articles received for publication [50]. Third, on the part of authors, the growing pressure to publish and obtain funding for research may be inducing the misconduct responsible for retraction. However, the use of funds for the financing of (subsequently) withdrawn studies constitutes an important element that further aggravates the responsibilities of authors guilty of fraudulent actions at scientific level. In our study, it was not possible to trace the amount of money funded in individual studies, but about 25% of the studies included resulted as supported by an external form of financing.

To date, the overall number of retractions represents a very small percentage (about 0.1%) of the volume of all publications in rehabilitation, and it represents about 10–15% of all the retractions in the 4 databases analyzed. At first sight, this appears to be a positive finding. However, these figures are difficult to interpret and compare with other scientific sectors, due to the differences in bibliometric index levels [51], selection criteria, or search strategies used by the authors. For example, in the recent review by Kardeş et al. [37] concerning the rehabilitation and sports sciences areas, the search was restricted only to the journals indexed in the Science Citation Index Expanded and Social Sciences Citation Index, yielding much narrower results than ours.

In the 2009–2018 period, the retraction rate increased significantly from a mean of 15.6 articles per 10,000 publications in 2009 to 26.6 in 2018 (Fig. 4). This means that the trend in the ratio of retracted articles to all publications is slightly but constantly increasing, and needs to be periodically monitored. However, it is not clear whether the increase of the retraction rate is a reflection of an increase in misconduct or of increased detection of flawed articles, or both. Known fraudulent publications are thought to be only a small part of the total number of fraudulent publications, and many cases have probably never been discovered [52]. Therefore, the actual number of articles withdrawn in rehabilitation is elusive and the data presented in this study may represent only the tip of the iceberg.

The areas more represented were musculoskeletal and sports, followed by neurology, orthopedic and gerontology. As expected, the most prevalent topic was rehabilitation (20%), with significant percentage of article retracted focusing also on drugs (17%), surgery (13%) and physical activity (13%). Due to the pressures to demonstrate efficacy/effectiveness of interventions, a higher percentage of RCTs might be expected. However, about one third of all retracted publications were observational studies. This is probably due to the lower costs necessary to conduct this type of study compared to an RCT, and to the greater ease of being published without having to go through a registration, for example. Overall, clinical studies accounted for more than half of all records, while reviews - with or without meta-analyzes - were about one-fifth.

It is interesting to note that two-thirds of all retracted publications were published in N-OA journals, and many of the retractions seem to be in renowned, well-known journals. This may reflect the greater attention of the editors and expert reviewers of these journals in identifying problems that may lead to retraction. The judgment is more complicated with regard to OA journals, for which it is necessary to make a clear qualitative distinction. Legitimate OA journals offer access to a wide variety of useful information at no cost, and constitute a great value for the readers. However, the perception that some OA journals may lead to a general decline in the quality of scientific publishing is well known, and the ease with which bogus information can be published in such journals defined as “predatory” has been well described by Cook et al. [53]. In rehabilitation, about one-third of articles withdrawn came from pure OA journals, but none of these was included in Jeffrey Beall’s list of predatory journals [54]. Since predatory journals are not indexed in the main databases, it is reasonable to hypothesize that the cases of misconduct are significantly greater than reported in this study, and the full picture will likely never emerge.

The consequences of treatment decisions based on misleading or inaccurate data can be easily understood. A very good illustration of the dangers of fraudulent scientific publication is the case of a retraction due to fabrication of the data of two trials aimed to study the effects of oral vitamin supplementation on prevention of fractures, authored by the same Japanese research group. These studies were included in subsequent reviews and meta-analyses, which resulted in retraction [55] or a warning notice published directly by the authors. For example, Torgerson [56] published a letter of explanation to alert readers to this concern, which resulted in no longer statistically significant results and changed the conclusions of the review study. However, these warnings were issued 9 to 12 years after publication, leading to a potential negative influence on patient management strategies and unnecessary expense for patients or healthcare systems.

### Limitations

Our study has several limitations. The retrospective analysis was limited to the articles available in four databases, and hence possibly underestimated the number of retractions, in particular in OA journals. Second, the selection of articles deemed to be of rehabilitation interest and therefore included in the study was subjective. To compensate for this bias, three independent reviewers evaluated the pertinence to rehabilitation of each publication. Third, it is possible that some retractions eluded the search queries, leading to a greater actual number of retractions than reported.

Finally, there could be many cases of fraud and research misconduct that are not picked up by editors and publishers and, therefore, the articles are never withdrawn. For these reasons, this analysis may have underestimated the true rate of retractions and scientific misconduct in the rehabilitation literature.

### Future implications

Being up to date is a priority for healthcare professionals, including physiotherapists. However, staying up to date also means being aware of falsified or error-generated data, and considering the implications at clinical level. Hence, the withdrawn of publications must be easily identifiable. The best way to correctly mark withdrawn publications has been debated, and solutions that do not require active and repetitive searches are thought to be more effective [57]. For example, CrossRef’s CrossMark initiative (http://www.crossref.org/crossmark/) helps to identify the latest version available for an article, in order to be sure that it has not been retracted or modified. Usually, withdrawn papers are clearly indicated as being retracted on journal websites. Regrettably, this is not always true of the downloadable PDF files, which do not adhere to the COPE guidelines in managing the full text.

Currently, the main purpose of retractions is to correct the literature and ensure its integrity rather than to punish authors who misbehave. However, a strategy to stem research misconduct could be to prosecute the authors responsible for it, prohibiting, for example, the publication of other manuscripts for a certain period of time, or through a pecuniary sanction, returning the funds received for the conduct of the study. This could be a deterrent to help prevent recurrence.

Finally, it may be helpful to have a specific database shared by the scientific journals containing all the retracted articles, so that the retractions and the responsible authors can be easily identified.

## Conclusions

The data presented in this study indicate that the number of retracted articles in rehabilitation is on an upward trajectory, suggesting that the scientific community is becoming more active in identifying suspect articles. However, retracted articles represent a very small percentage (about 0.1%) of the overall volume of publications in rehabilitation. Given the potential for harm that arises from withdrawn articles, this low percentage is to be welcomed, but it may not represent the true prevalence of misconduct due to the high number of journals not indexed in the databases consulted. In any case, physiotherapists must beware of the dangers of misleading information that can come from withdrawn publications.

## Supplementary Information

**Additional file 1.** Key terms and search strategies used in each database.

**Additional file 2.** Journals that were found to have issued retraction notices.

## Data Availability

Not applicable.

## Abbreviations

COPE: Committee on Publication Ethics
PRISMA: Preferred Reporting Items for Systematic Reviews and Meta-Analyses
WoS: Web of Science
OA: Open Access
N-OA: Non-Open Access
SD: Standard deviation
IQR: Interquartile range
