# Supplementary material for: Retracted articles in rehabilitation: just the tip of the iceberg? A bibliometric analysis
Source: Arch Physiother. 2020 Nov 30;10:21. doi: 10.1186/s40945-020-00092-w (PMC7706289; doi:10.1186/s40945-020-00092-w)
Supplement: Supplementary file 1 — Additional file 1. Key terms and search strategies used in each database. [file 40945_2020_92_MOESM1_ESM.docx]

**Additional file 1**

**Key terms and search strategies used in each database**

**PubMed**

Search strategy**:** (“Rehabilitation"[Mesh] OR "Exercise Therapy"[Mesh] OR "Telerehabilitation"[Mesh] OR "Neurological Rehabilitation"[Mesh] OR "Stroke Rehabilitation"[Mesh] OR "Cardiac Rehabilitation"[Mesh] OR "Physical and Rehabilitation Medicine"[Mesh]) OR ("Physical Therapy Modalities"[Mesh] OR "Physical Therapy Specialty"[Mesh]) OR ("Musculoskeletal Manipulations"[Mesh] OR "Musculoskeletal Diseases"[Mesh] OR "Musculoskeletal Pain"[Mesh] OR "Postural Balance"[Mesh] )

Filters applied: Article Type: Retracted publication/Retraction of publication

**RetractionWatch**

No specific keyword was used in the search strategy. The database was searched by applying the following two filters: Subjects: Health Sciences (HSC) - ‘Medicine - Rehabilitation/Therapy’, and Nature of Notice: ‘Retraction’

The Version 1.0.6.0 of the database was used.

**Web of Science**

The database was searched by using the terms ‘Retraction of’ in the Title section, and refined by setting the Web Of Science Categories to ‘Rehabilitation’, and the Document Types to ‘Retraction or correction’ tags.
Timespan: All years

**Wikiletters**

Simple terms were searched within WK-Retracted branch as follows: Acupuncture # Amputee # Amputation # Ankle # Arthritis # Balance # Behaviour # Calf # Cervical # Coordination # Dry-Needling # Eccentric # Edema # Education # Elbow # Electromyography # Electrotherapy # Exercise # Fitness # Foot # Frailty # Gait # Hand # Head # Hip # Hydrotherapy # Impaired # Incontinence # Isometric # Joint # Knee # Lumbar # Manipulation # Manual Therapy # Massage # Mobilization # Motor # Movement # Muscular # Musculoskeletal # Muscle # Neck # Neurodevelopmental Therapy # Neurofacilitation # Orthoses # Osteoporosis # Pain # Palsy # Pelvis # Perineum #Physical Activity # Physical Therapy # Physiotherapy # Postural # Posture # Rehabilitation # Respiratory # Sacro-Iliac # Scoliosis # Shock Wave # Shoulder # Spine # Splint # Stretching # Strength # Tai Chi # Tape # Taping # Telerehabilitation # Temporomandibular # Therapy # Thigh # Thoracic #Training # Ventilation # Whiplash # Wrist
